# Microstructure and Hot Deformation Behavior of Twin Roll Cast Mg-2Zn-1Al-0.3Ca Alloy

**DOI:** 10.3390/ma12071020

**Published:** 2019-03-28

**Authors:** Kristina Kittner, Madlen Ullmann, Thorsten Henseler, Rudolf Kawalla, Ulrich Prahl

**Affiliations:** Institute of Metal Forming, Technische Universität Bergakademie Freiberg, Bernhard-von-Cotta-Straße 4, 09599 Freiberg, Germany; madlen.ullmann@imf.tu-freiberg.de (M.U.); thorsten.henseler@imf.tu-freiberg.de (T.H.); rudolf.kawalla@imf.tu-freiberg.de (R.K.); ulrich.prahl@imf.tu-freiberg.de (U.P.)

**Keywords:** twin roll casting, magnesium alloy, calcium, Mg-Zn-Al-Ca alloy, texture, flow curve, processing map

## Abstract

In the present work, the microstructure, texture, mechanical properties as well as hot deformation behavior of a Mg-2Zn-1Al-0.3Ca sheet manufactured by twin roll casting were investigated. The twin roll cast state reveals a dendritic microstructure with intermetallic compounds predominantly located in the interdendritic areas. The twin roll cast samples were annealed at 420 °C for 2 h followed by plane strain compression tests in order to study the hardening and softening behavior. Annealing treatment leads to the formation of a grain structure, consisting of equiaxed grains with an average diameter of approximately 19 µm. The twin roll cast state reveals a typical basal texture and the annealed state shows a weakened texture, by spreading basal poles along the transverse direction. The twin roll cast Mg-2Zn-1Al-0.3Ca alloy offers a good ultimate tensile strength of 240 MPa. The course of the flow curves indicate that dynamic recrystallization occurs during hot deformation. For the validity range from 250 °C to 450 °C as well as equivalent logarithmic strain rates from 0.01 s^−1^ to 10 s^−1^ calculated model coefficients are shown. The average activation energy for plastic flow of the twin roll cast and annealed Mg-2Zn-1Al-0.3Ca alloy amounts to 180.5 kJ/mol. The processing map reveals one domain with flow instability at temperatures above 370 °C and strain rates ranging from 3 s^−1^ to 10 s^−1^. Under these forming conditions, intergranular cracks arose and grew along the grain boundaries.

## 1. Introduction

Twin roll casting (TRC) of magnesium alloys becomes more important due to the economic and energy efficient process of manufacturing sheets and strips compared to conventional thin sheet production. Sheets of magnesium wrought alloys, produced via twin roll casting, exhibit good mechanical properties [1]; however, the main drawback concerning their application potential is their low formability at low temperatures [2]. The formability of magnesium wrought alloys is closely related to the resulting microstructure, texture and hcp lattice structure. Improving formability, through grain refinement and texture modification, can be obtained by different approaches: thermomechanical processing [3,4], severe plastic deformation such as equal-channel angular extrusion (ECAE) [5], differential speed rolling (DSR) [6] or cross-rolling (CR) [7] and chemical alloying. Processes of severe plastic deformation may be detrimental to cost development and limitations in possible shapes and dimensions. Studies about chemical alloying recommend rare earth (RE) elements [8,9] as well as Ca [10,11,12], Sr [13,14] or Li [15,16] being preferred to influence the texture modification, towards weaker basal or random texture development. Apart from their enhanced formability compared to conventionally available alloys, fabrication of novel alloys via twin roll casting has scarcely been published yet. Park et al. [2] demonstrate that twin roll casting of a ZAX400 alloy leads to the formation of an equiaxed dendrite microstructure with no occurrence of inverse segregations, which adversely affect the mechanical properties and surface quality of the strip. Wang et al. [17] observed columnar dendrites at the surface and a transition to equiaxed dendrites in the mid-thickness of an AM31 + 0.2 Ca alloy after twin roll casting. Main microstructural features are in accordance with those offered by other magnesium wrought alloys [1]. So far, most of previous works are focused on the conventional Mg alloys (AZ or ZK systems). However, systematic research on the hot deformation behavior of fine-grained Mg-Zn-(Al)-Ca alloy is rarely reported. In a study by Tong et al. [18] about the compressive deformation behavior of the as-extruded and as-ECAPed Mg–5.3Zn–0.6Ca (wt%) alloys at 200 °C to 300 °C, the compression behaviors of both conditions were mainly dominated by climb-controlled dislocation creep through grain boundary diffusion. The as-ECAPed alloy presented a lower activation energy (100–108 kJ/mol instead of 160–172 kJ/mol for as-extruded alloy), which might be derived from non-equilibrium grain boundaries [18]. Kulyasova et al. [19] investigated the application of high-pressure torsion (HPT), which leads to the formation of ultrafine-grained structures in Mg-Zn-Ca with an average grain size of 150 nm. HPT processing and additional annealing at 200 °C results in a high ultimate tensile strength due to grain refinement and dispersion hardening; whereas, the retention of a good ductility (8.5%) is conditioned by the activation of dislocation slip in non-basal planes [19]. There are also publications dealing with RE elements addition in the Mg-Zn-Ca alloy. For example, Qi et al. [20] studied the hot compression behavior and processing characteristics of Mg-3Zn-0.3Ca-0.4La (wt.%). The results suggested that deformation parameters had significant effects on the deformation behaviour and dynamic recrystallization. The average activation energy of plastic deformation was calculated to be 188.9 kJ/mol and might be attributed to the existence of precipitates and a fine microstructure [20].

The magnesium alloy Mg-2Zn-1Al-0.3Ca (ZAX210) serves as investigation material of the present work. In recent years, the ZAX210 alloy has gained importance, due to its advantageous strength and formability because of Ca addition. Current studies are concerned, among other research, with casting and hot rolling of the ZAX210 alloy as well as the resulting microstructure, texture and mechanical properties [21,22,23]. Hoppe et al. [23] revealed a texture of the cast and hot rolled ZAX210 sheet exhibiting two peaks along the rolling direction as well as a basal pole spread in transverse direction. The authors assume that this texture evolution due to Ca is responsible for the improved formability. Letzig et al. [24] show microstructure, texture and mechanical properties of ZAX210 sheets after twin roll casting and hot rolling. The sheets offer a fine grained and homogenous microstructure and a texture similar to the RE-containing alloy ZE10. Twin roll casting and hot rolling of ZAX210 are also part of the studies of Ullmann et al. [25]. The authors conclude that the good formability of the ZAX210 sheets is due to the weakening of the texture, which originates from the recrystallization process during hot rolling were Ca induces particle stimulated nucleation.

The processing map technique based on the dynamic material model (DMM) has been widely used to understand the workability of materials [26]. With help of the map, the description of the hot forming behavior and the determination of the most reasonable thermal processing parameters is practicable. 

The present work is focused on twin roll casting and the resulting microstructure, texture and mechanical properties as well as the hot deformation behavior of the ZAX210 alloy. These apparitions are associated with the hot workability (evaluated by processing maps) of the TRC and annealed state. The hot forming behavior is the first important component in the understanding of the entire process chain for the production of a good formable Mg alloy.

## 2. Materials and Methods 

The magnesium alloy with a nominal composition (wt.%) ZAX210 was subjected to a twin roll caster (Institute of Metal Forming, Technische Universität Bergakademie Freiberg, Saxony, Germany) on industrial scale. The chemical composition of the investigated alloy is listed in Table 1. Ingots of the ZAX210 were melted in a steel crucible (Institute of Metal Forming, Technische Universität Bergakademie Freiberg, Saxony, Germany) at 730 °C under protective gas atmosphere, transferred into a preheated casting system and twin roll cast. The thickness of the TRC sheets was approximately 5.3 mm and is further referred to as the TRC state. Subsequently, the TRC sheets were annealed at different temperatures ranging from 340 °C to 460 °C for holding times ranging from 2 h to 12 h. After annealing, continuous plane strain compression tests at three different strain rates (0.1 s^−1^, 1 s^−1^, 10 s^−1^) and temperatures (250 °C, 350 °C, 450 °C) were performed on a servo-hydraulic hot deformation simulator (Institute of Metal Forming, Technische Universität Bergakademie Freiberg, Saxony, Germany). The specimen size was 20 mm × 30 mm × 5.3 mm and deformed by a 6-mm-wide carbide tool insert perpendicular to the TRC direction up to equivalent logarithmic strains equal to 1. As lubricant, a liquid graphite-oil-mixture was used. Based on the recorded force-displacement data isothermal flow curves were calculated. Here, divergences due to friction at the tool interface as well as softening effects due to an increase in temperature from dissipated forming energy have been corrected numerically. To guarantee a sufficient degree of statistical certainty, five comparative samples of each forming condition were tested.

Samples of the TRC material were prepared for the characterization of the microstructure. Specimens for optical microscopy were prepared by conventional grinding and polishing with oxide polishing suspension (OPS), followed by etching in a solution of ethanol, glacial acetic acid, picric acid and distilled water. Optical and scanning electron microscopy (SEM, Institute of Material Science, Technische Universität Bergakademie Freiberg, Saxony, Germany) were used to study microstructural characteristics. The chemical composition of the individual structural constituents was measured by energy dispersive X-ray spectroscopy (EDX, Institute of Material Science, Technische Universität Bergakademie Freiberg, Saxony, Germany) and X-Ray Diffraction (XRD) analysis on a 10 mm × 5.3 mm cross section of the TRC strip. XRD was performed on a Seifert-FPM RD7 (Institute of Material Science, Technische Universität Bergakademie Freiberg, Saxony, Germany) using CuKα radiation (λ = 1.540598 Å). Diffraction patterns were recorded within the 2Θ-range of 20° to 150°. The step size was chosen at 0.02° and a step time of 25 s.

Tensile tests were conducted at room temperature. Samples were extracted in TRC direction (0°) according to DIN 50125. The specimen shape H with an initial measuring length of 80 mm was selected. Tensile tests were performed on the universal tensile testing machine AG-100 (Institute of Metal Forming, Technische Universität Bergakademie Freiberg, Saxony, Germany) with a crosshead speed of 2 mm/min. 

Pole figures of the sheets in TRC as well as the annealed condition were calculated from electron backscatter diffraction (EBSD). The specimens were polished by ion beam for 2 h with a voltage of 4 kV and 2 mA beam current. The EBSD analysis was performed using a FEI Versa 3D scanning electron microscope (Academic Centre for Materials and Nanotechnology, University of Science and Technology, Krakow, Poland) equipped with a Hikari EBSD detector. The voltage used for acquisition of EBSD data was 15–20 kV, and the step size was 0.65 µm. All orientation maps were processed with the EDAX/TSL OIM Data collection software version 7 using a batch processing operation. For analysis of the EBSD data, calculation of ODF (orientation distribution function) and pole figures the free MTEX MATLAB toolbox [27] was used. 

The preparation of the processing maps is based on thermodynamic analysis of the flow curves. Further information about processing maps based on DMM derivation can be found in [28]. The method considers the complementary relationship between the rate of heat generation induced by the forming process and the rate of energy dissipation associated with microstructural changes such as recovery and dynamic recrystallization (stability domains) and material damage (instability domains). To represent the power dissipation due to microstructural mechanism, a non-dimensional efficiency index *η* is used, see Equation (1) [26], where *m* is the strain rate sensitivity (function of forming temperature and strain rate). The changes of *η* on the temperature-strain rate field create the processing map. In addition to the *η* contours, an instability parameter $\xi \left(\dot{\varepsilon }\right)$ given first by Prasad et al. [26] described by the Equation (2) is applied to delineate the temperature-strain rate regimes of flow instability on the processing map.

(1) $$\eta \;=\;\frac{2m}{m+1}$$

(2) $$\xi (\dot{\varepsilon })\;=\;\frac{\partial \ln\left(\frac{m}{m+1}\right)}{\partial \ln\dot{\varepsilon }}+\;m\;\leq \;0$$

## 3. Results

### 3.1. Microstructure and Texture of the Twin Roll Cast ZAX210 Alloy

**Figure 1:**
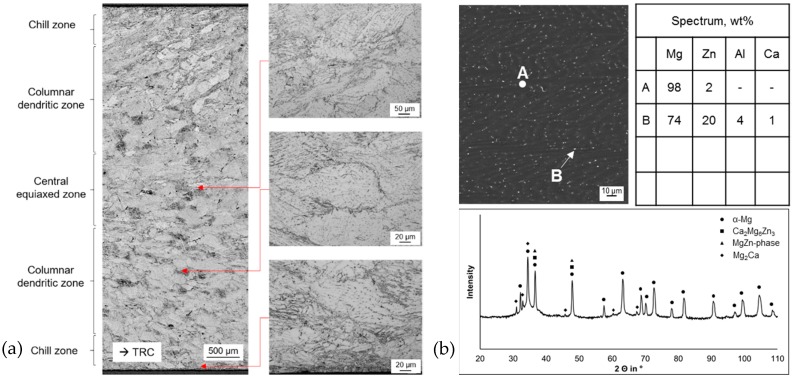
(**a**) Microstructure of the TRC (twin roll cast) state ZAX210 [25], divided into three characteristic sections through thickness cross-section and (**b**) scanning electron micrograph, results of the XRD analysis (X-ray diffraction) and chemical composition of structural constituents according to marking in SEM (scanning electron microscope) image determined via EDX analysis (energy dispersive X-ray spectroscopy) for the ZAX210 in TRC condition [30].

Twin roll casting of ZAX210 leads to the formation of the characteristic microstructure, which develops during TRC and is well investigated for the magnesium alloy AZ31 [1,29]. Due to the superimposed solidification and forming processes, the alloy exhibits an inhomogeneous microstructure [25]. The morphology of the microstructure is significantly influenced by the processing parameters. The inhomogeneous microstructure of the TRC strip can be divided into three zones (chill zone, columnar dendritic zone and central equiaxed zone) with different characteristics (Figure 1a). During solidification coarse columnar and severely deformed dendrites develop, which also tend to cause the occurrence of unwanted artificial twins in optical micrographs due to sample preparation. The dendrites grow in opposite direction to the heat flow of the melt, predominantly perpendicular to the surface of the rolls. The rolling leads to an inclination of the columnar dendrite with respect to the neutral axis of the TRC strip. Within the chill and the central equiaxed zone, fine grains occur because of constitutional undercooling or dynamic recrystallization during hot deformation.

The interdendritic areas as well as a small segregation line in the middle of the strip reveal intermetallic compounds. Results of the XRD analysis indicate different kinds of intermetallic compounds (Mg_2_Ca, MgZn-phases and Ca_2_Mg_6_Zn_3_), but predominantly α-magnesium was detected (Figure 1b). Numerous measurements were carried out, nevertheless, due to the small size of the intermetallic compounds (<1 µm), further investigations with greater resolution are necessary to back these results as the surrounding α-magnesium matrix possibly influenced the measurement.

**Figure 2:**
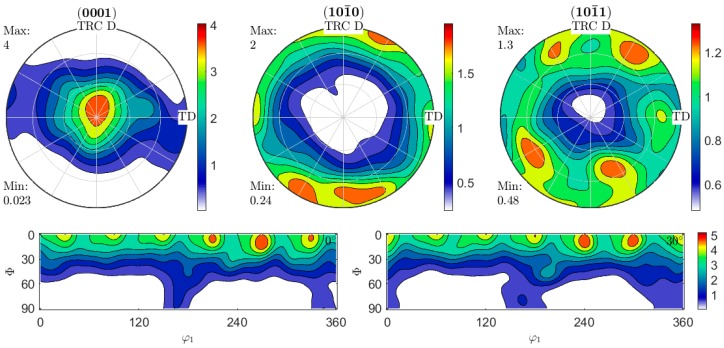
(0001)-basal, $\left(10\bar{1}0\right)$-prismatic and $\left(10\bar{1}1\right)$-pyramidal pole figures related to the TRC direction and transverse direction of the TRC strips denoted as TRC D and TD (transverse direction) respectively as well as calculated ODF sections at *φ*_2_ = 0° and 30°.

The pole figures of TRC state ZAX210 presented in Figure 2 indicate the formation of a basal texture with a slight spread along TD. $\left(10\bar{1}0\right)$ and $\left(10\bar{1}1\right)$ pole figures reveal preferential orientations of the pyramidal and prismatic planes. The peaks in the $\left(10\bar{1}0\right)$ and $\left(10\bar{1}1\right)$ pole figures indicates specific alignment of the unit cells around the c-axis. The angles of these peaks correspond to the a-axes angles of the unit cell as also recognizable in the illustrated ODF sections, and show a slight rotation about the c-axis. Just as in the basal pole figure, this rotation might be caused by a sloped specimen surface.

### 3.2. Microstructure and Texture of the Annealed ZAX210 Alloy

**Figure 3:**
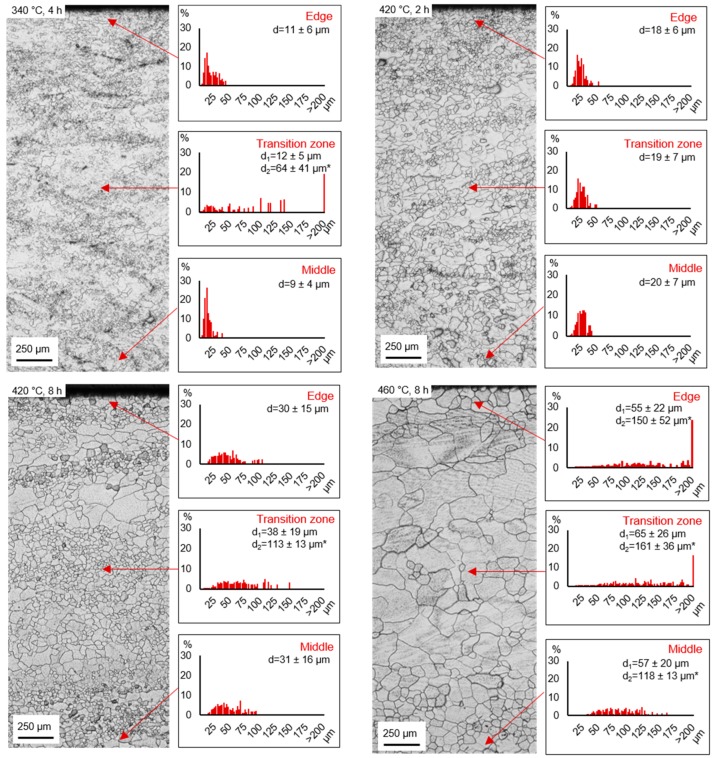
Microstructures of the annealed ZAX210 alloy with regard to the heat treatment conditions (340 °C, 4 h; 420 °C, 2 h; 420 °C, 8 h and 460 °C, 8 h) and associating grain size distribution depending on the characteristic section of the TRC strip (edge, transition zone and middle); diagrams show grain size class vs. percentage of the grain area in relation to the total area of all measured grains (→ TRC direction, * two maxima due to bimodal grain size distribution).

Heat treatment was performed in order to ensure homogenization and the development of a microstructure with equiaxed grains. Resulting microstructures and grain size distributions measured by linear intercept method are summarized in Figure 3. During heat treatment of TRC strip, the rearrangement of the microstructure starts at 340 °C. However, areas of the initial microstructure remain surrounded by small recrystallized grains. With increasing temperature, the initial microstructure diminishes and recrystallization proceeds. Thus, heat treatment at 420 °C and a holding time of 2 h results in a rearranged microstructure offering fine equiaxed grains with an average grain size ranging from approximately 18 µm at the edge to approximately 20 µm in the middle of the strip (Figure 3, 420 °C, 2 h). An increasing temperature or holding time (Figure 3, 420 °C, 8 h and 460 °C, 8 h) leads to anomalous grain growth. Consequently, a bimodal grain size distribution arises with an average exceeding 100 µm.

**Figure 4:**
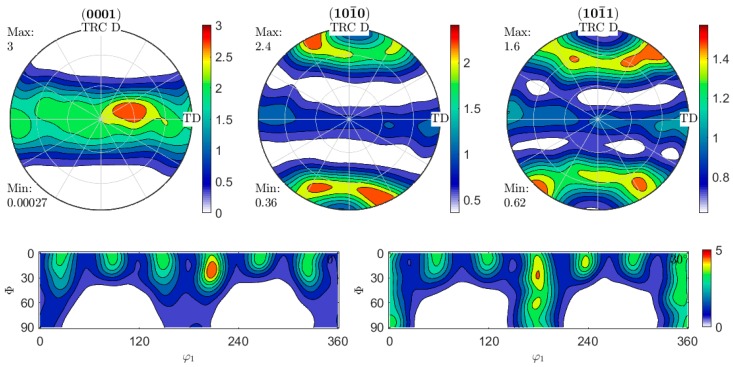
(0001)-basal, $\left(10\bar{1}0\right)$-prismatic and $\left(10\bar{1}1\right)$-pyramidal pole figures taken from TRC D–TD plane of the annealed (420 °C, 2 h) strip as well as calculated ODF sections at *φ*_2_ = 0° and 30°.

Figure 4 shows the texture of the annealed condition (420 °C, 2 h). Compared to the TRC condition, a strong spreading of the c-axis towards TD from ND occurred. As it can be seen from the $\left(10\bar{1}0\right)$ and $\left(10\bar{1}1\right)$ pole figures the prismatic and pyramidal poles reveal a similar alignment with TD. This prismatic and pyramidal pole spread originates from the basal pole spread, but also indicates a majority arrangement of the unit cells with parallel a-axes. As seen in the illustrated ODF sections, the pre-existing preference has remained, but here too the intensities have decreased because of the basal pole spread.

### 3.3. Mechanical Properties at Room Temperature

**Figure 5:**
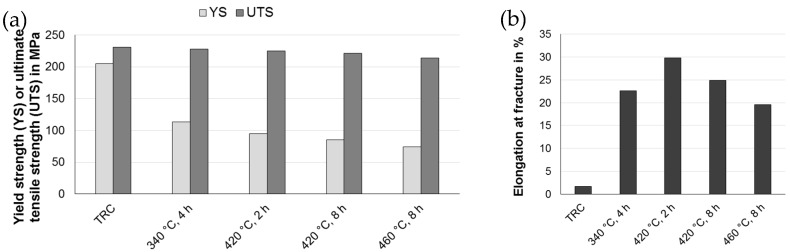
Mechanical properties at room temperature of the ZAX210 alloy after twin roll casting and different heat treatments: (**a**) yield strength and ultimate tensile strength and (**b**) elongation at fracture.

In TRC state, the ZAX210 alloy offers values of 205 MPa yield strength and 231 MPa ultimate tensile strength. Due to the inhomogeneous microstructure, which arises during twin roll casting because of solidification kinetics, the elongation at fracture is diminished. Other magnesium alloys, for example AZ31, AM50 or WE43, show similar results [1]. Heat treatment and the accompanying processes of static recrystallization lead to a significant improvement of the elongation at fracture. Depending on homogeneity of the microstructure as well as grain size and grain size distribution the elongation at fracture ranges from ~20% (460 °C, 8 h) to ~30% (420 °C, 2 h). The enhancement of the ductility is accompanied by a decrease in yield strength. During heat treatment dislocation climbing and annihilation takes place as well as precipitates are dissolved and consequently softening of the material occurs and the yield strength decreases (Figure 5). Increasing temperature or holding time of the annealing results in a slight decrease of the ultimate tensile strength. 

### 3.4. Hot forming Behavior of Twin Roll Cast and Annealed ZAX210 Alloy

**Figure 6:**
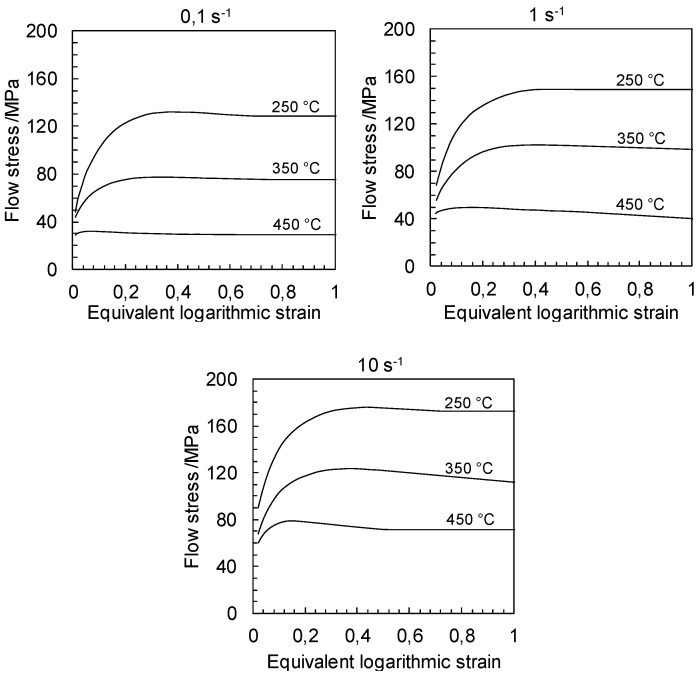
Exemplary flow curves of TRC and annealed ZAX210 alloy during plane strain compression at temperatures of 250 °C–450 °C and strain rates of 0.1 s^−1^–10 s^−1^.

Exemplary flow curves during plane strain compression at temperatures of 250 °C, 350 °C and 450 °C as well as strain rates of 0.1 s^−1^, 1 s^−1^ and 10 s^−1^ are shown in Figure 6. The flow curves exhibit smooth hardening and softening phases, while the maximum flow stress value moves–together with the strain rate increase–towards greater equivalent logarithmic strain values. The flow stresses decrease when the strain rate is decreased or the temperature is increased. The flow stress initially increases because of strain hardening and reaches a peak value, subsequently decreasing with further strain. The course of the flow curves indicate that dynamic recrystallization occurs. The rates of strain hardening and strain softening vary with the deformation conditions. The initial hardening is associated with the increase in dislocation density. At lower strain rates, dislocation multiplication is less rapid and contributes to a lower strain hardening effect than that at higher strain rates. Deformation at higher temperatures or at lower strain rates result in higher dynamic recrystallization kinetics because of higher diffusivity at higher temperatures and more time for nucleation and growth of dynamically recrystallized grains.

## 4. Discussion

### 4.1. Texture Evolution of the Twin Roll Cast and Annealed ZAX210 Alloy

Twin roll casting results in the formation of a basal texture, where a slight tilt of the peak can be seen. Preferred orientations and strong basal textures usually arise during rolling, when slip mainly occurs on basal planes [31]. Typically, TRC strips (for example AZ31 alloy) exhibit a low basal texture due to the directional growth of columnar grains [32]. Results of the influence of a heat treatment on the resulting microstructure of a TRC state ZAX210 in comparison to other magnesium alloys are shown in [25]. The texture of the TRC strip originates from the rolling deformation, which is associated with the TRC process. Similar results were reported for TRC of AZ31 alloy in [32] and [33]. According to Soomro et al. [34], the cause of possible tilting is due to the formation of twins. It is reported that twinning as an important deformation mode in magnesium alloys can alter the orientation of original grains and consequently {10–12} tensile twins may be responsible for the basal pole tilt. Because of the low critical resolved shear stress (CRSS) of about 2.5 MPa [34] tensile twins can easily arise. Nevertheless, twinning was not observed in the present TRC strip. In contradiction to the literature the authors presume, that due to the high temperatures that occur, prismatic and pyramidal slip is also part of the dominating deformation mechanisms leaving little contribution due to twinning, as can be presumed from the alignment in the $\left(10\bar{1}0\right)$-prismatic and $\left(10\bar{1}1\right)$-pyramidal pole figures in annealed state. Further investigations are required in order to clarify the dominant mechanism for basal pole tilting during TRC. Because of the specific microstructure, which results from the combination of casting and rolling during TRC process, further heat treatment has an important effect on the microstructure as well. 

During heat treatment of TRC strip the rearrangement of the microstructure is combined with softening processes, which already occur at low temperatures (Figure 3, 340 °C). The stored deformation energy, introduced during TRC, which is characterized by forming with minor reduction (log. strain < 0.1), is sufficient to initiate static recrystallization during heat treatment. In previous studies static recrystallization has been proposed to be related to particle-stimulated nucleation (PSN), shear band induced nucleation (SBIN) and deformation twin induced nucleation (DTIN). The XRD results of the TRC condition imply the occurrence of Mg_2_Ca precipitates, which can act as nucleation sites for recrystallization. The addition of calcium contributes to the formation of the Mg_2_Ca phase [35,36]. XRD results reveal, that precipitates have dissolved during heat treatment at 460 °C, because only α-magnesium matrix was detected. Very small particles are able to impede grain growth by inhibition of grain boundary movement. The heat treatment at temperatures above 420 °C may lead to their dissolution and consequently grain growth occurred [37,38]. Zimina et al. [39] revealed, that after a homogenization treatment of a TRC AZ31 strip tilting of the grains towards TRCD occurred and led to the balancing of the texture along the cross-section. The above-mentioned recrystallization mechanisms are known to result in a decrease of the basal texture intensity as well as in the development of more randomized textures [40,41]. Assuming, that PSN is the dominant recrystallization mechanism, randomly oriented nuclei were provided and a weaker recrystallization texture arose. Here are parallels to the displayed textures in TRC and annealed state recognizable. According to the results of Sandlöbes et al. [42] Ca addition is correlated with a significant decrease of the intrinsic stacking fault energy, which is connected with the ductility increase by the increased activity of pyramidal <c + a> dislocations.

### 4.2. Hot Deformation Behavior of Twin Roll Cast and Annealed ZAX210 Alloy

#### 4.2.1. Analysis of Hot Compressive Deformation Behavior

**Figure 7:**
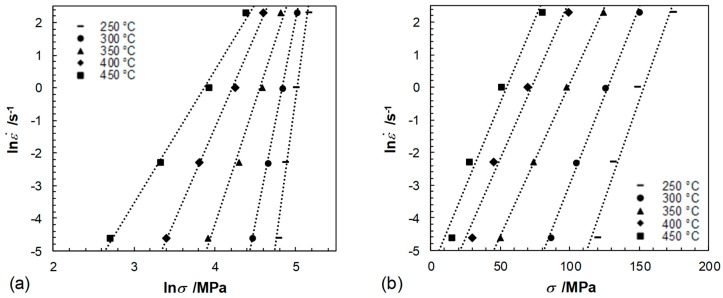
Relationship between ln$\dot{\varepsilon }$ and (**a**) ln*σ* (**b**) *σ*, temperature dependent.

**Figure 8:**
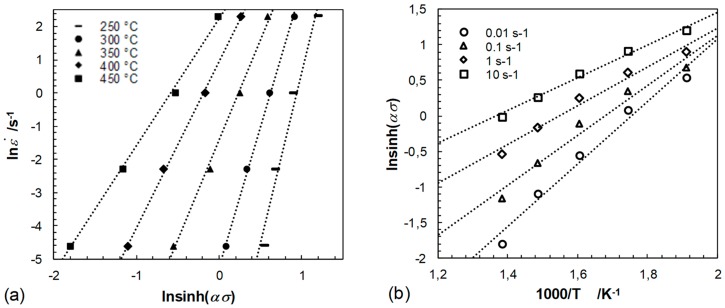
Relationship between (**a**) ln$\dot{\varepsilon }$−ln[sinh(*ασ*)] at different temperatures and (**b**) ln[sinh(*ασ*)]^−1^/T as a function of the tested strain rates.

**Figure 9:**
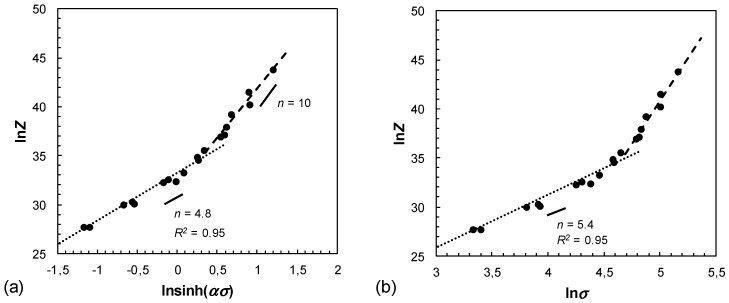
Relationship between Zener–Hollomon parameter and peak stress in hot compression test, (**a**) ln*Z*−ln[sinh(ασ)] and (**b**) ln*Z*−lnσ.

By assessing the values of the power dissipation efficiency *η* and the instability parameter $\xi \left(\dot{\varepsilon }\right)$ in dependency of temperature, logarithmic strain as well as strain rate in a processing map, the characteristics of plastic flow are evaluated. The power law relationship of Sellars and Tegart’s [43,44] was used to derive the deformation mechanisms during hot deformation. The relationship is expressed as:

(3) $$\dot{\varepsilon }=\;A\;{\left[\sin h\left(\alpha \sigma \right)\right]}^{n\;}\exp\left(-\frac{Q}{R\cdot T}\right)$$

where $\dot{\varepsilon }$ is the strain rate (s^−1^), *A* is the material constant, *n* is the stress exponent, *α* is a fitting parameter, *σ* is the peak stress (MPa), *Q* is the average activation energy for plastic flow (kJ/mol), *R* is the gas constant (8.314 J/(mol·K)) and *T* is the thermodynamic deformation temperature (K). The parameters of this equation were obtained from the mean values of the slopes of graphs relating flow stress to strain rate and temperature in linear dependence, see Figure 7, Figure 8 and Figure 9, and are as follows: $A=3.463\cdot 10^{15},n=4.8$, *α* = $0.0109\;{\mathrm{MPa}}^{-1}$, *Q* = 180.5 kJ/mol. The calculated model coefficients have validity for temperatures ranging from 250 °C to 450 °C as well as for equivalent logarithmic strain rates of 0.01 s^−1^ to 10 s^−1^. The activation energy *Q* indicating deformation difficulty degree during hot deformation depends on concurrent dynamic precipitate, dislocation pinning effects and the second phase. The high *Q* value (higher than that for lattice diffusion of magnesium, which is 135 kJ/mol) might be attributed to the existence of precipitates and the fine microstructure. Precipitates could hinder dislocation movement during the deformation and exerted a resistant force for lattice self-diffusion. Additionally, the fine grains provide more grain boundaries acting as barriers to the dislocation movement and lattice self-diffusion. Besides, the addition of alloying elements presumably have an effect on the activation energy, due to the effect of solution strengthening. 

As is well known, the Zener–Hollomon parameter *Z* is determined by *Z* = $\dot{\varepsilon }$·exp(*Q*/(*RT*)). The dependence of ln*Z* to ln[sinh(ασ)] is shown in Figure 9. The interpretation of the power law relationship depends on the fitting of the relationship between the Zener–Hollomon parameter and ln[sinh(ασ)]. At low *Z* values linear regression was determined to an accuracy of (*R^2^*) = 0.95, revealing a good fit of the hyperbolic sine function. Following the constitutive equation

(4) $${Z\;=\;A\;\sin h}^{n}\left(\alpha \sigma \right)$$

**Figure 10:**
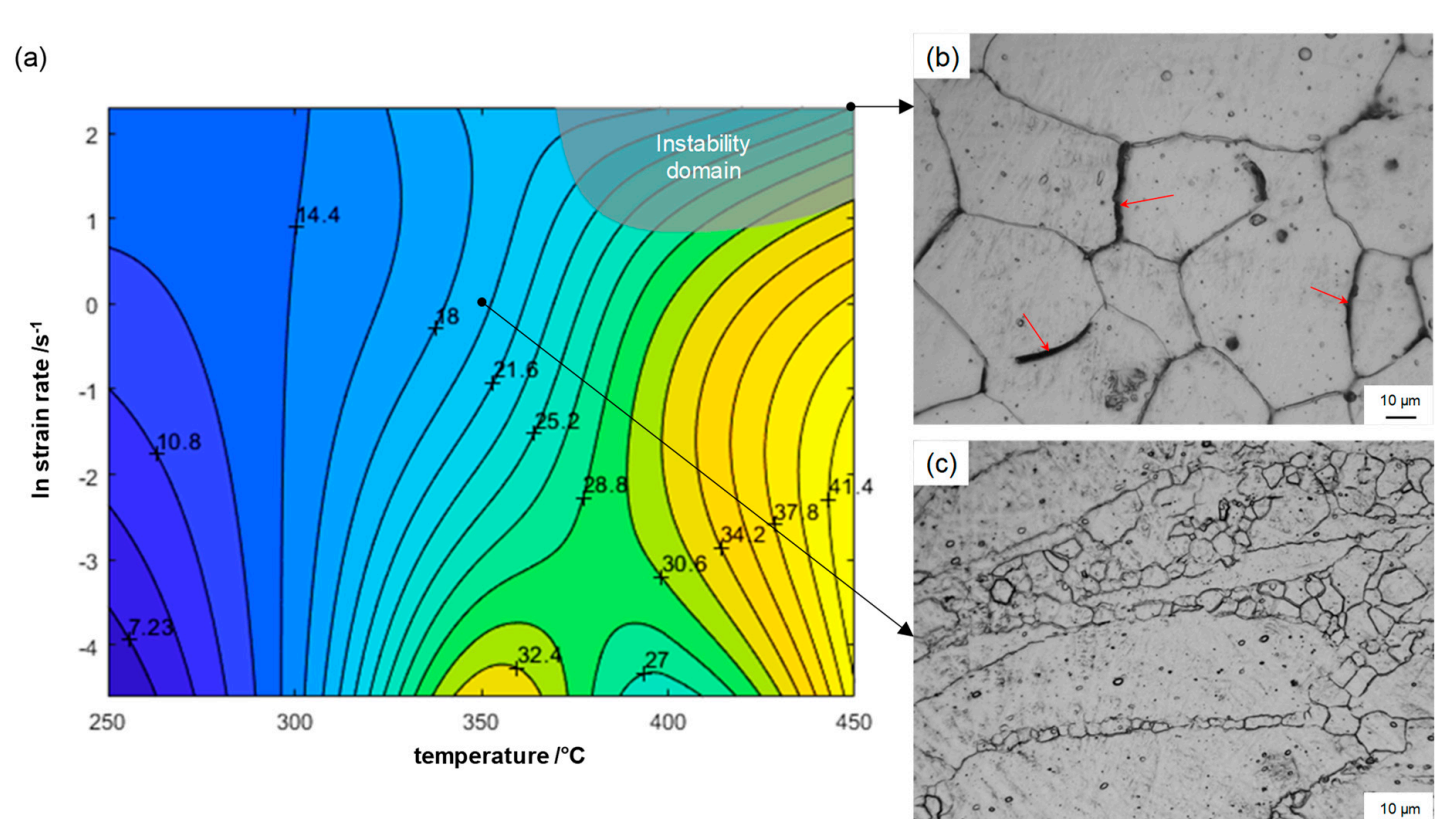
(**a**) Processing map for TRC and annealed ZAX210 at an equivalent logarithmic strain of 0.4 and (**b**) microstructure within the instability domain (450 °C; 10 s^−1^) and (**c**) resulting microstructure in stable domain (350 °C; 1 s^−1^).

The stress exponent *n* at low *Z* values (low strain rates and high temperatures) is 4.8. Under consideration of the results from [45] that dislocation climb creep corresponds to *n* > 5 and solute drag creep to *n* = 3, the presented results let assume that dislocation climb creep is the dominant mechanism of plastic flow. Increasing ln*Z* values results in a deviation from linearity, due to the power law breakdown (PLB). PLB arises at high strain rates and low temperatures and is characterized by *n* = 10. It is recognized that shear banding occurs in the PLB regime. At high strain rates there is not enough time available for dislocations to rearrange. Consequently, dislocations are not able to sweep out excess vacancies which arise during plastic deformation [46]. Figure 9a shows the plot of ln*Z*−ln[sinh(ασ)], corresponding to an activation energy *Q* = 180.5 kJ/mol and the fitting parameter *α* = 0.0109 MPa^−1^. In comparison to the curves in Figure 9b, an improved degree of linearity can be observed as expected from the characteristic hyperbolic sine function. However, with increasing Z values, linearity decreases resulting in the appearance of instability domains in the processing map (Figure 10). This result indicates that the data in the instability regime are not valid within the PLB regime, described by Equation (4).

#### 4.2.2. Processing Map

The processing map for TRC and annealed ZAX210 alloy at a logarithmic strain of 0.4, which is representative in a typical rolling pass, is shown in Figure 10a. The contour numbers denote percent power dissipation efficiency *η* and the shaded domains regions of flow instability $\xi \left(\dot{\varepsilon }\right)$ < 0. The processing map exhibits one domain with flow instability at temperatures above 370 °C and strain rates between 3 s^−1^–10 s^−1^. The instability, which arises at high strain rates is attributed to localized shear [26]. The microstructure after deformation at 450 °C and 1 s^−1^, which refers to the instability region of the processing map, is shown in Figure 10b. Under these forming conditions, intergranular cracks arose and grew along the grain boundaries. Consequently, the instability region is undesirable for processing and hence should be avoided. Although the resulting microstructure in Figure 10c has different grain sizes, it has corrugated grain boundaries with some fine-grained nucleation sites. These microstructural characteristics are suitable for hot forming processing such as rolling, extrusion and forging.

It should be noted that the processing maps are sensitive to the initial condition like chemical composition and processing history, resulting in different microstructures with different forming behavior. The hot workability of Ca-containing alloys like Mg-4Al-2Ba-2Ca [47], Mg-4Al-2Ba-2Ca [48], Mg-1Zn-1Ca [49] and Mg-3Sn-2Ca-0.4al-0.4Zn [50] has been investigated by developing processing maps, which indicate that the alloys are workable at temperatures between 300 °C and 500 °C at low strain rates. At strain rates above 0.1 s^−1^ and 1 s^−1^ instability regions occur, except for the area between 340 °C and 380 °C. Due to the different chemical composition and process history of the alloy described in this paper, various deviations may occur when comparing the microstructure, texture as well as the hot working behavior.

## 5. Conclusions

The presented work focused on twin roll casting and the resulting microstructure and texture, mechanical properties as well as the hot deformation behavior of the Mg-2Zn-1Al-0.3Ca alloy. Findings obtained permit the following conclusions:

1. Twin roll casting leads to the formation of a characteristic microstructure, which can be divided into the three sections: chill zone, columnar dendritic zone and central equiaxed zone. Within the chill and the equiaxed zone fine grains occur as a result of constitutional undercooling or dynamic recrystallization during hot deformation.

2. Twin roll casting results in the formation of a weakly pronounced basal texture, which can be characterized by a slight asymmetric tilting of the basal pole. A preferential orientation by the prismatic or pyramidal poles is assumed in alignment with the twin roll casting direction. 

3. During heat treatment of twin roll cast strip the rearrangement of the microstructure is combined with softening processes, which already occur at low temperatures (340 °C). The stored energy introduced during twin roll casting, which is characterized by forming with minor reduction (log. strain < 0.1), is sufficient to initiate static recrystallization during heat treatment. Heat treatment at 420 °C and a holding time of 2 h lead to a homogeneous microstructure consisting of fine equiaxed grains as a result of nucleation and grain growth. Due to the occurrence of Mg_2_Ca phases in the TRC strip, particles stimulated nucleation is assumed being the dominant recrystallization mechanism.

4. After heat treatment a stronger spreading of the c-axis of towards TD occurred. Assuming, that PSN is the dominant recrystallization mechanism, randomly oriented nuclei were provided and a weaker recrystallization texture arose.

5. The features of the flow curves indicate that dynamic recrystallization occurs during hot deformation. At lower strain rates, dislocation multiplication is less rapid and contributes to a lower strain hardening effect than that at higher strain rates. Deformation at higher temperatures and/or at lower strain rates can result in higher dynamic recrystallization kinetics because of higher diffusivity at higher temperatures and more time for nucleation and growth of dynamic recrystallization. Thus, a lower strain hardening effect is observed at higher temperatures and lower strain rates.

6. The average activation energy for plastic flow amounts to 180.5 kJ/mol for the twin roll cast and annealed Mg-2Zn-1Al-0.3Ca alloy. The stress exponent *n* at low *Z* values (low strain rates and high temperatures) is 4.8, suggesting that dislocation climb creep dominates the plastic flow.

7. The processing map at an equivalent logarithmic strain of 0.4 exhibit one domain with flow instability at temperatures above 370 °C and strain rates between 3 s^−1^–10 s^−1^. Under these forming conditions, intergranular cracks arose and grew along the grain boundaries. Consequently, the instability region is undesirable for processing and hence should be avoided.

## Figures and Tables

**Table 1:** Chemical composition of the ZAX210 alloy (wt.%).

Zn	Al	Ca	Mn	Cu	Fe	Ni	Others	Mg
2.290	0.920	<0.250	0.040	0.001	0.005	0.001	<0.045	Bal.
